# Establishment and Evaluation of a Stable Bovine Thyroid Cell Line for Investigating Foot-and-Mouth Disease Virus

**DOI:** 10.3389/fmicb.2018.02149

**Published:** 2018-09-11

**Authors:** Ruoqing Mao, Dehui Sun, Fan Yang, Hong Tian, Zixiang Zhu, Haixue Zheng, Xiangtao Liu

**Affiliations:** State Key Laboratory of Veterinary Etiological Biology, National Foot and Mouth Diseases Reference Laboratory, Key Laboratory of Animal Virology of Ministry of Agriculture, Lanzhou Veterinary Research Institute, Chinese Academy of Agricultural Sciences, Lanzhou, China

**Keywords:** bovine thyroid, cell line, foot-and-mouth disease virus, immunity, *in vitro* model

## Abstract

Foot-and-mouth disease virus (FMDV) has a wide host range. Its pathogenesis varies among hosts and types of viruses. Most investigations of pathogenesis have been performed on cattle and swine. However, FMDV research in cattle is hampered by the lack of a stable *in vitro* infection model. In this study, the stable bovine thyroid (BTY) cell line hTERT-BTY from primary BTY cells was established by telomerase reverse transcriptase over expression. The results of karyotype analysis and experiments on morphological and biological characteristics indicated that this cell line possessed the qualities of primary BTY cells, which could be extended indefinitely with stable morphology and steady growth rates. The hTERT-BTY cell line, has 60 chromosomes including 29 pairs of autosomes and 1 pair of sex chromosomes without structure aberrations. It can express thyroid-specific function genes thyroid-stimulating hormone receptor and sodium/iodide symporter in high abundance ratios. The cell line is sensitive to FMDV strains and is expected to be used as a powerful tool for FMDV clinical diagnosis, separation, detection and culture. Also, the different mRNA expression levels in infected and uninfected hTERT-BTY cells were analyzed in this study to identify the pathways of immunity using RNA-seq. The results suggested that the hTERT-BTY cell line could be regarded as an effective tool for the immune response exploration of FMDV. In conclusion, this study provided a useful tool for FMDV clinical diagnosis, separation, detection, and culture. The cell line also could serve as an *in vitro* model to study the mechanism underlying FMDV pathogenicity and host–virus interaction.

## Introduction

The foot-and-mouth disease virus (FMDV) belongs to the Aphthovirus genus of the *Picornaviridae* family. This virus causes an acute vesicular disease in domestic and feral cloven-hoofed animals, which is characterized by the appearance of erosions and vesicles on hairless skin and cutaneous mucosae (Mason et al., 2003; Grubman and Baxt, 2004). Although the disease is associated with low mortality, outbreaks can cause global impact and huge economic losses through direct effects on international trading and agriculture food security (Knight-Jones and Rushton, 2013). Suckling mice and cell lines isolated from hamster kidney (BHK-21) and swine kidney (PK-15, IB-RS-2, and SK-6) are the most successful experimental model systems for FMDV separation, culture, assay, and research (Gagliardi et al., 1966; De Castro, 1970; Kasza et al., 1972). Relative sensitivity to different virus strains varies among these systems, which can cause virus mutation via culture of the virus across host species. In contrast, the most sensitive system for FMDV detection, isolation, and culture is a primary monolayer culture of bovine thyroid (BTY) cells, which are 100- to 1,000-fold more sensitive to various unmodified bovine FMDV strains compared with the aforementioned systems (Snowdon, 1966; Brehm et al., 2009). However, primary BTY cells cannot be passaged stably or frozen with remaining sensitivity. During the process of research and diagnosis, these preparations of primary BTY cells requires the sacrifice of a bovine fetus to obtain thyroid tissues, which is at odds with the original intention of “animal welfare” and is both time-consuming and laborious. Therefore, a cell line with the qualities of primary BTY cells needs to be established to facilitate FMDV separation, culture, assay, and research.

Foot-and-mouth disease virus has a wide host range and the clinical symptoms of FMD differ among hosts. Pathogenesis has been investigated mainly in cattle and swine (Arzt et al., 2011). Cattle are the most susceptible. Some studies have suggested that the initial site of viral replication in infected cattle is the pharynx (Burrows et al., 1981), whereas the cells of the esophageal-pharyngeal (OP) region have a key role in FMDV-persistent infection (Zhang et al., 2013). Most fundamental research concerning the pathogenesis of FMD and FMDV host–virus interaction is done *in vivo*. However, FMDV research in cattle has been hampered by the lack of a stable *in vitro* infection model. The investigations on pathogenic mechanisms, especially the research on immune response, need a stable cell line and the pathway of the immunity should be revealed at the cellular level. Therefore, a stable bovine cell line with the characteristics of primary BTY cells, which can be used in FMDV diagnostic and fundamental research, is urgently needed. In this study, a bovine thyroid cell line (hTERT-BTY) was established and evaluated using a series of approaches. Finally the transcriptome RNA-seq was adopted to reveal the bioinformatics of FMDV host–virus interaction. The typical genes related to the immune response were partly selected to verify the results of RNA-seq using real-time quantitative PCR (q-PCR). The results suggested that this cell line might serve as a tool or an *in vitro* model to separate, culture, and assay FMDV and also to study FMDV host–virus interaction.

## Results

### Morphological and Biological Characteristics of Primary BTY and hTERT-BTY Cells

**FIGURE 1:**
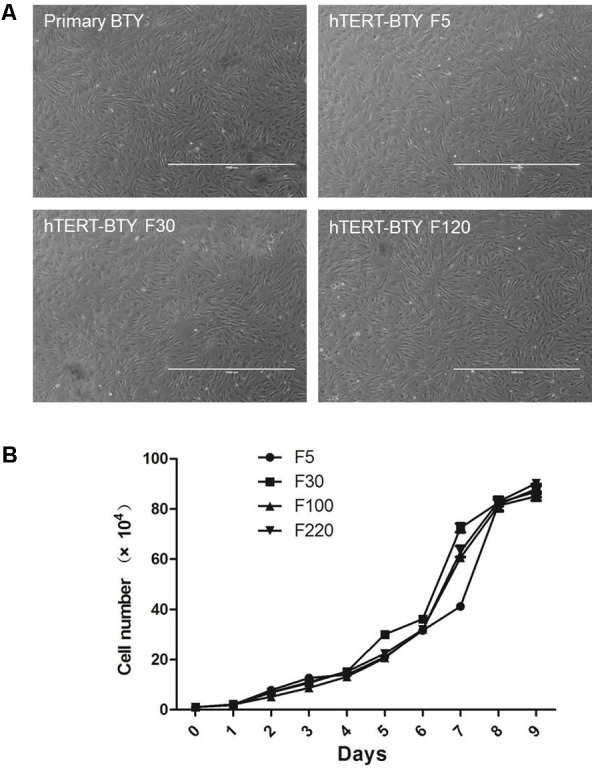
Morphologic and growth curves of hTERT-BTY cells. **(A)** Confluent monolayer formed by primary BTY cells and subcultured hTERT-BTY cell line at passages 5, 30, and 120. **(B)** hTERT-BTY cells at passages 5, 10, 100, and 220 were seeded onto 24-well plates at a density of 1 × 10^4^ cells/well. The cells were trypsinized every 24 h starting from 1 to 9 days after seeding and quantified using a hemocytometer. Experiments were performed in triplicate and repeated three times with similar results. Data are represented as mean ± SD (*n* = 3).

Primary BTY cells were isolated from BTY tissues to establish an immortalized BTY cell line. Previous studies confirmed that the shortening of telomeres was the reason for cell senescence in most normal somatic cells. Therefore, the over-expression of the telomerase reverse transcriptase (TERT) gene and activation of telomerase are critical strategies in cellular immortality (Levy et al., 1992; Li et al., 2012). The hTERT expression plasmid was transduced into primary BTY cells and the G418-resistant colonies were isolated. One colony was expanded and cultured, this colony was named hTERT-BTY (Invention Patent, China, ZL201410421962.4. CCTCC, C2014109). Confluent monolayers of the hTERT-BTY cells at passages 10–100 complied with typical primary BTY cell morphology was shown in **Figure 1A**. Growth curves of hTERT-BTY cells at passages 5, 10, 100, and 220 were created (**Figure 1B**). The cell line showed a steady growth rate in passages 5–220, whereas the viability of the primary BTY cells began to decline after passage 6. Taken together, the results suggested that the hTERT-BTY cell line could be extended indefinitely with steady morphology and growth rate.

### Expression of Thyroid-Specific Genes

**FIGURE 2:**
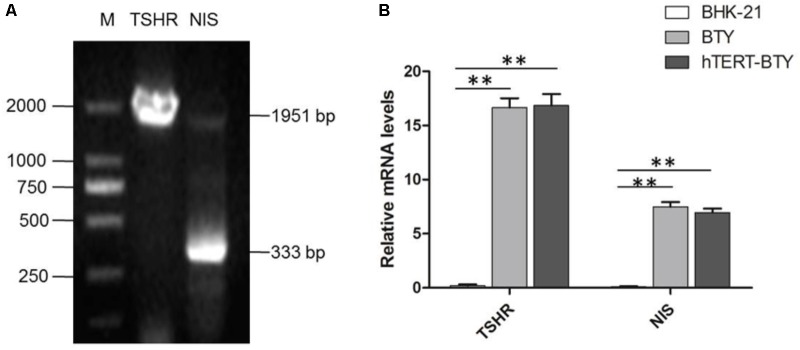
Tissue-specific gene expression of hTERT-BTY cells. **(A)** hTERT-BTY cells at passage 30 were detected using PCR and nucleic acid electrophoresis. M, marker; TSHR, the PCR products of bovine THSR (1.951 kb) gene segment; NIS, the PCR products of bovine NIS (333 bp) gene segment. **(B)** Expression of TSHR and NIS of BHK-21, primary BTY, and passage-220 hTERT-BTY cells was detected using q-PCR. Data are represented as mean ± SD (*n* = 3). Statistical significance was analyzed by Student’s *t*-test: ^∗^*P* < 0.05, ^∗∗^*P* < 0.01.

Thyroid-stimulating hormone receptor (TSHR), thyroglobulin, thyroid peroxidase and sodium/iodide symporter (NIS) are thyroid-specific function genes (Szinnai et al., 2007), the mRNA expression levels of which are the main indexes of terminally differentiated thyrocytes (De Felice and Di Lauro, 2004). Total RNA from hTERT-BTY cells at passage 30 was analyzed to determine whether hTERT-BTY cells expressed the typical genes inherent to primary BTY cells, so as to verify whether the hTERT-BTY cell line still maintained the genetic characteristics of primary BTY cells, The hTERT-BTY cells could express TSHR and NIS in high abundance ratios (**Figure 2A**). The PCR products were checked by sequencing, and the sequence alignment of the genes between hTERT-BTY cells and primary BTY cells was performed. The amino acid sequence homology of both TSHR and NIS was 100%. To confirm the conclusion, the expression of TSHR and NIS gene in BHK-21, primary BTY cells and hTERT-BTY cells at passage 220 was detected by q-PCR. As shown in **Figure 2B**, TSHR and NIS were expressed in high levels in primary BTY and hTERT-BTY cells compared with BHK-21 cells. The results indicated that the hTERT-BTY cell line maintained the main features of primary BTY cells.

### Chromosomal Analysis

**FIGURE 3:**
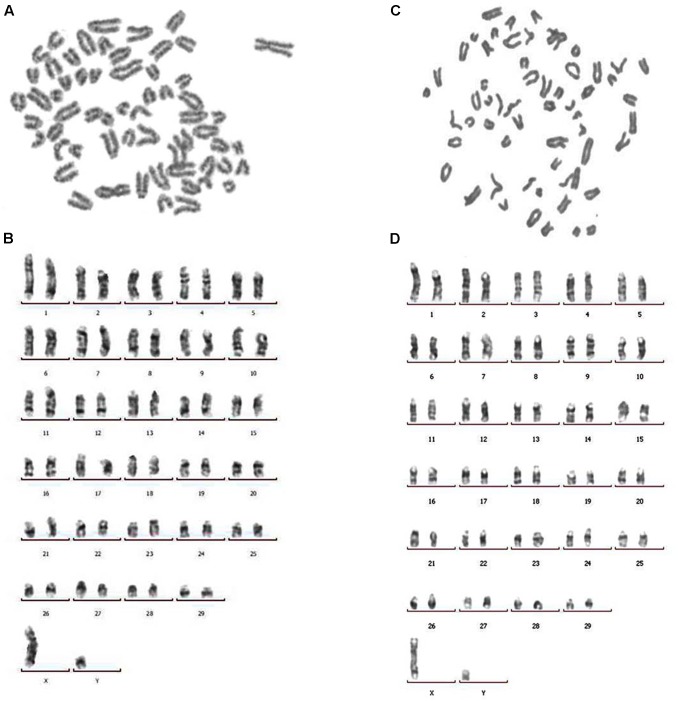
Chromosomal analysis of primary BTY cells and the hTERT-BTY cell line. **(A)** Chromosomes of primary BTY cells with a diploid number of 60. **(B)** GTG-banded primary BTY cell karyotype. **(C)** Chromosomes of hTERT-BTY cell line with a diploid number of 60. **(D)** GTG-banded hTERT-BTY cell line karyotype.

Both structural and number variations in chromosomes were found using karyotype tests, indicating genetic changes associated with cell cancerization. Chromosome numbers of primary BTY cells and hTERT-BTY cells at passage 30 were analyzed. As shown in **Figures 3A,C**, the number of chromosomes in the hTERT-BTY cell line did not change. Both primary BTY cells and hTERT-BTY cells have 60 chromosomes, including 29 pairs of autosomes and 1 pair of sex chromosomes. The G-banding technique was used to confirm whether the process of immortalization induced structural aberrations of hTERT-BTY cells. The results were analyzed on the basis of the bovine GTG-banded standard karyotype (Iannuzzi, 1996). All chromosomeswereunchanged in the hTERT-BTY cells, and the cell line had the same karyotype as the primary BTY cells (**Figures 3B,D**).

### Growth Characteristics of FMDV

**FIGURE 4:**
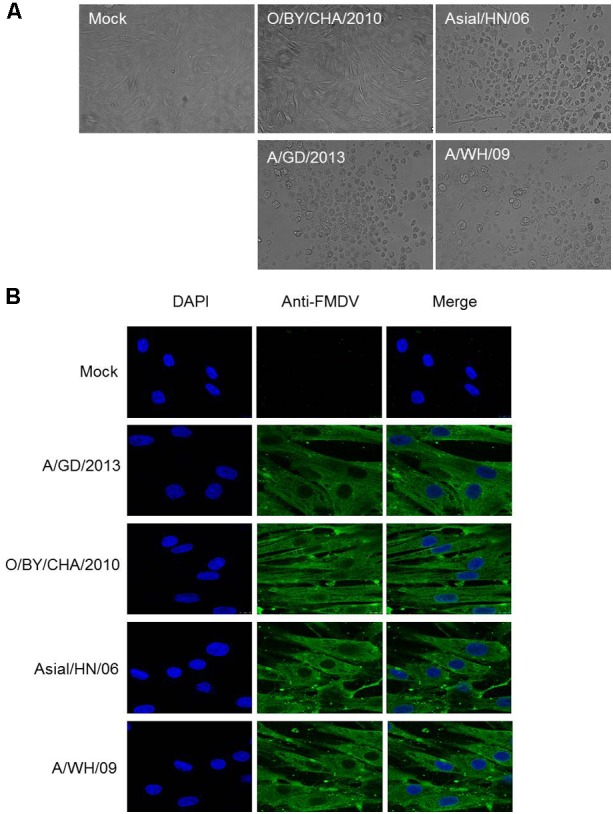
Growth characteristics of FMDV strains in hTERT-BTY cells. **(A)** CPE caused by O/BY/CHA/2010, Asial/HN/06, A/GD/2013, and A/WH/09 at MOI 0.001 on the passage-30 hTERT-BTY cell line at 72 hpi. **(B)** Immunofluorescence assay of hTERT-BTY cells infected with FMDV. hTERT-BTY cells (2 × 10^5^ cells/well) were infected with or without O/BY/CHA/2010, Asial/HN/06, A/GD/2013, and A/WH/09 at a MOI of 0.001. The distribution of FMDV (green) was observed using confocal microscopy 24 h after the infection. hpi, hours post infection.

**FIGURE 5:**
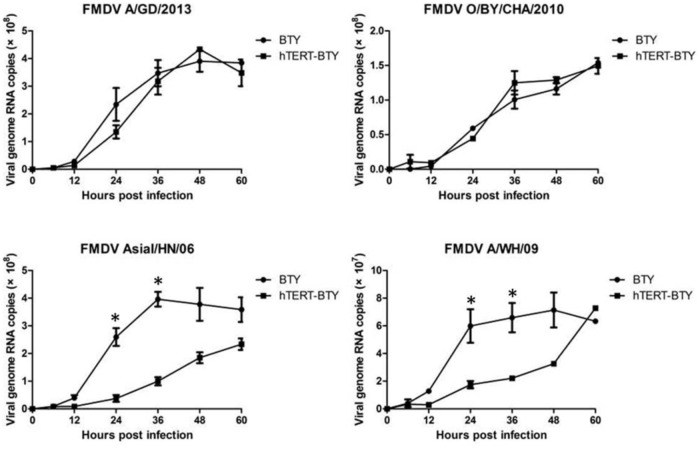
Viral genome RNA replication dynamics. hTERT-BTY cells (5 × 10^5^ cells/well) were infected with the indicated four viruses at a dose of 1 × 10^6^ viral genome RNA copies for 1 h, and unabsorbed viruses were washed. Cells and supernatants were harvested 0, 6, 12, 24, 36, 48, and 60 h after the infection, and the expression of viral RNA was determined using RT-PCR. Experiments were performed in triplicate and repeated three times with similar results. Data are represented as mean ± SD (*n* = 3). Statistical significance was analyzed by Student’s *t*-test: ^∗^*P* < 0.05, ^∗∗^*P* < 0.01.

Cytopathic effect (CPE) induced by Asial/HN/06, O/BY/CHA/2010, A/GD/2013, and A/WH/09 strain was observed, and virus multiplication curves were determined to detect whether hTERT-BTY cells were sensitive to FMDV. FMDV infection in hTERT-BTY cells at passage 50 was detected by the development of CPE, as shown in **Figure 4**. FMDV strains containing Asial/HN/06, GD/2013 and A/WH/09 could induce clear CPE. However, the induction of CPE by the O/BY/CHA/2010 strain at a multiplicity of infection (MOI) of 0.001 was not observed 72 h post infection (**Figure 4A**). Immunofluorescence experiments were carried out to further visualize the distribution of FMDV in hTERT-BTY cells. As illustrated in **Figure 4B**, hTERT-BTY cells were infected by all the four FMDV strains, and the viruses were mainly distributed in the cytoplasm. The titers of O/BY/CHA/2010, A/GD/2013 and A/WH/09 in hTERT-BTY cells, BTY cells at passage 3 and BHK-21 cells were determined by median tissue culture infected dose (TCID50) assay to further demonstrate the susceptibility of hTERT-BTY cells to FMDV. The results showed that the titers of all the three FMDV strains in hTERT-BTY cells were as high as that in other cells (**Table 1**). Then, total viral RNA of FMDV-infected BTY cells at passage 3 and hTERT-BTY cells at passage 50 was detected using qRT-PCR. The results suggested that the number of RNA copies of the four FMDV strains mentioned above increased acutely from 6 h post infection and reached a peak in 48–60 h in hTERT-BTY cells. The trends of viral genome RNA replication dynamics of O/BY/CHA/2010 and A/GD/2013 in hTERT-BTY cells were consistent with those in passage-3 BTY cells, with the exception of the two A-type FMDV strains, where the replication levels were slightly lower than those in passage-3 BTY cells (**Figure 5**). The results suggested that the indicated four FMDV strains could replicate in hTERT-BTY cells. All the results indicated that the hTERT-BTY cell line supported the sustainable propagation of O-, A-, and Asia I-type FMDV strains.

**Table 1:** Viral titers in different host cells.

FMDV	LogTCID_50_/1 mL		
	
	hTERT-BTY	BTY	BHK-21
O/BY/CHA/2010	8.6 ± 0.21	8.6 ± 0.33	8.0 ± 0.20
Asial/HN/06	8.1 ± 0.29	8.2 ± 0.10	8.0 ± 0.25
A/GD/2013	8.75 ± 0.23	8.6 ± 0.19	8.33 ± 0.25
A/WH/09	8.2 ± 0.40	8.3 ± 0.27	7.77 ± 0.31


### Differentially Expressed Genes Between FMDV-Infected and Uninfected hTERT-BTY Cells

**FIGURE 6:**
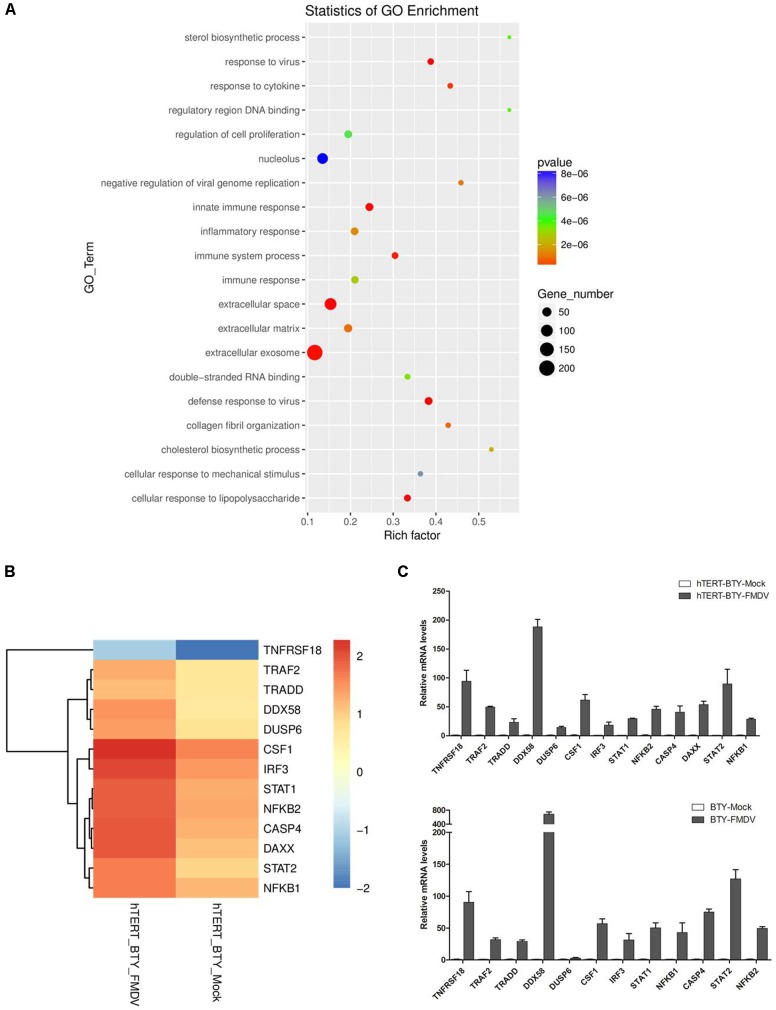
Differentially expressed genes between FMDV-infected and uninfected hTERT-BTY cells. **(A)** hTERT-BTY cells were infected with O/BY/CHA/2010 at an MOI of 0.01. The transcriptome RNA-seq and scatterplots of significant enrichment items of GO analyses associated with differentially expressed genes were compared between FMDV-infected and uninfected hTERT-BTY cells. The vertical axis is the GO term, and the horizontal axis is a rich factor of GO. The *P*-value denotes the significance of the GO term (the recommended cutoff of *P*-value is 0.05). **(B)** Heatmap of selected differentially expressed genes involved in immune response. **(C)** Validation of the differentially expressed genes using the qPCR assay in hTERT-BTY cells and BTY cells at passage 3. Experiments were performed in triplicate and repeated three times with similar results. Data are represented as mean ± SD (*n* = 3).

Differentially expressed genes involved in multiple responses in hTERT-BTY cells infected with FMDV or not were collected and analyzed to explore whether the hTERT-BTY cells could be regarded as an effective tool for FMDV research. The clean dates were obtained after stringent quality check and filtering. A total of 17,300 genes were detected and analyzed, of which 1,309 were identified (*P* < 0.05). Compared with the uninfected control cells, 921 genes were differentially upregulated and 388 genes were downregulated in the FMDV-infected cells. The differentially genes, including 97 (23.83%) immune-related genes participating in 8 immune-regulatory processes, were up to 407 and involved in 25 biological processes (**Figure 6A**).

The q-PCR analysis was employed to determine the reproducibility of the differential gene expression so as to confirm and verify the transcriptome analysis results. The immune response-related genes *CASP4, NF-κB, IRF3, DDX58, TRADD, TRAF2, STAT1, STAT2, CF1, TNFRSF1B*, and *DAXX* were randomly selected for analysis and comparison between FMDV-infected and uninfected hTERT-BTY cells or BTY cells at passage 3. As shown in **Figures 6B,C**, the results of q-PCR corresponded with transcriptome analysis results.

## Discussion

In recent decades, a series of cell lines, including BHK-21 (Gagliardi et al., 1966), IB-RS-2 (De Castro, 1970), SK-6 (Kasza et al., 1972) and PK-15 (Luo et al., 2011), were established as important tools for FMDV separation, culture, assay and research. Further, the fetal goat tongue cell line ZZ-R 127 was confirmed to be highly sensitive to FMDV (Brehm et al., 2009). However, no bovine cell line is available for bovine FMDV *in vitro* research. Primary BTY cells with high sensitivity to cattle virus have been found (Plowright et al., 1960; Snowdon, 1966; O’Donnell et al., 2014). In this study, the stable BTY cell line hTERT-BTY was established using primary BTY cells obtained from a freshly slaughtered 1-day-old healthy Holstein calf. This cell line was subcultured to passage 220 and still possessed highly stable growth activity. Primary cells derived from non-cancerous tissues had a limited lifespan when they were cultivated *in vitro*. The technique used to immortalize primary BTY cells in this study was the overexpression of TERT. Previous studies confirmed that the shortening of telomeres was the reason for cell senescence in most normal somatic cells. Telomerase could increase the length of telomeres and inhibit cellular senescence. Therefore, the over expression TERT gene and activation of telomerase are critical strategies in cellular immortality (Levy et al., 1992; Li et al., 2012). This method was able to extend the lifespan of primary cells and immortalize primary cells without causing phenotypic or genotypic changes or cell cancerization (Duncan et al., 2000; Lee et al., 2004; Ramboer et al., 2014). However, other methods for primary cell immortalization always altered the cell characteristics.

Many supplements, including thyroid-stimulating hormone (TSH), bovine insulin, transferrin, and L-glutamine, were added to the culture medium to promote cell proliferation and avoid differentiation as previously described (Curcio et al., 1994). A previous study showed that TSH stimulated primary cultures of human thyroid cells (Kogai et al., 2000; Petitfrere et al., 2001). In this study, TSH was essential for the adherence and proliferation of the hTERT-BTY cell line and the hTERT-BTY cells lost adhesive capacity when the medium lacked bovine TSH.

A series of indexes of biological characteristics, including morphology, growth rate, and tissue-specific gene expression, were noted in the hTERT-BTY cell line. In the present study, the cell line showed a stable growth rate. TSHR and NIS are thyroid-specific function genes, the mRNA expression levels of which are main indexes of terminally differentiated thyrocytes (Szinnai et al., 2007). TSHR and NIS genes were strongly expressed in hTERT-BTY and primary BTY cells compared with BHK-21 cells. In short, the hTERT-BTY cell line maintained the biological characteristics of primary BTY cells and exhibited a high level of proliferation.

Cattle have 60 chromosomes, including 29 pairs of autosomes and one pair of sex chromosomes (Bhambhani and Kuspira, 1969; Khatun et al., 2011). The number and structural errors of autosomal or sex chromosomes have a negative influence on cell performance. The karyotype text can find changes in the number of chromosomes and locate structural aberrations in chromosomes (Iannuzzi, 1996). G-, R-, C-, T-, and Q-banding is commonly used for chromosomal staining. G-banding can produce a visible karyotype by staining condensed chromosomes with Giemsa stain (Gustavsson and Hageltorn, 1976). It is important to ensure that the hTERT-BTY cell line maintains a karyotype similar to that of primary BTY cells. In this study, karyotype tests of chromosomal analysis and G-banding showed that the chromosome number of hTERT-BTY cells is accordance with primary BTY cells and without structural aberrations. These results indicated that the hTERT-BTY cell line was of bovine somatic cell origin without cancerization.

More importantly, this study found that the sensitivity of hTERT-BTY cell line to FMDV seemed close to that of primary BTY and BHK-21 cells. CPE caused by Asia-l/HN/06, A/GD/2013 and A/WH/09 at MOI 0.001 were observed at 24 hpi. However, CPE induced by the O/BY/CHA/2010 strain was not observed at 72 hpi. The results of viral replication experiment indicated that the four viruses could infect and proliferate in the hTERT-BTY cell line. The number of RNA copies of Asial/HN/06, O/BY/CHA/2010, A/GD/2013, and A/WH/09 in the hTERT-BTY cell line increased abruptly from 6 hpi and reached a relatively high peak. This mirrored similar trends with the FMDV strains in passage-3 BTY cells. Why some FMDV strains can proliferate in the cell line without causing CPE is still unknown and needs further investigation. All of the results given above showed that the cell line was sensitive to FMDV and could be used as a powerful tool for FMDV clinical diagnosis, separation, detection, and culture. Another important characteristic of FMDV is that it can reside persistently in host cells and produce a non-cytocidal infection. The tissues in the pharyngeal area of ruminants have an important role during persistent FMDV infection (O’Donnell et al., 2014; Pacheco et al., 2015). The mechanism underlying persistent infection is unclear, and the research has been hampered for the lack of an *in vitro* model. Benefiting from the characteristic that FMDV can proliferate in the hTERT-BTY cell line without causing CPE, the hTERT-BTY cell line is also expected to be as a research model of FMD-persistent infection.

The transcriptome analysis was performed using the NGS technology to evaluate the hTERT-BTY cell line for investigating FMDV. The differentially expressed genes correlated with the viral replication observed in hTERT-BTY. The results indicated differential expression of 1,309 genes between infected and non-infected hTERT-BTY cells. The cellular component and molecular function were enriched based on the finding that 20 kinds of GO or Pathway terms belonged to biological processes. The result suggested that hTERT-BTY modified its biological characteristics to defend FMDV. The present study found that the differential gene expression observed after FMDV infection resulted from the altered expression of genes involved in transcription and immune-related regulation. The altered expression of genes involved in transcription, cytokine and chemokine production, inflammation, apoptosis, and immune response were the significant reasons for the observed regulation in pathogenicity. It revealed the mechanism of interaction between FMDV and host from bovine. The 13 genes, including NF-κB1, NF-κB2, CASP4, IRF3, DDX58, TRADD, TRAF2, STAT1, STAT2, CSF1, TNFRSF18, DAXX, and DUSP6, were selected to confirm the findings of q-PCR. The gene NF-κB activity was significantly enhanced in swine cells inoculated with mutant FMDV (Zhu et al., 2010). However, FMDV infection could induce the degradation of NF-κB from swine (Alexandersen et al., 2003). The present study found that the NF-κB expression was upregulated after FMDV infection. TRAF2 reflected the extent of activity of the transcription factor NF-κB. DDX58, IRF3, NFKB1, NFKB2, STAT1, and STAT2 were involved in innate immunity. DAXX, DUSP6, CASP4, and TRADD were involved in apoptosis. The aforementioned results provided insight into the pathogenic mechanisms associated with FMDV infection in the bovine cell line.

## Conclusion

In conclusion, a stable BTY cell line named hTERT-BTY was established. The genetic, physical and biological characterizations of the cell line were also studied. FMDV could infect the cells and replicate at a high level. However, some specific FMDV strains could proliferate in the cells without causing CPE. This study provided a useful tool for FMDV clinical diagnosis, separation, detection, and culture. The cell line might also serve as an *in vitro* model to study the mechanism of FMDV replication, persistent infection, and host–virus interaction.

## Materials and Methods

### Specimen Preparation

Specimens from bovine throat tissues [thyroids and part of the weasand (esophagus)] were obtained from a freshly slaughterhouse slaughtered 1-day-old healthy Holstein calf. The tissue specimens were isolated and transferred to the laboratory in sterile phosphate-buffered saline (PBS) supplemented with 100 μg/mL streptomycin and 100 U/mL penicillin on ice within 30 min.

### Thyroid Isolation and Primary Culture of Bovine Thyroid (BTY) Cells

The specimens were washed several times with cold PBS to remove excess blood andthen placed into 70% (v/v) ethanol for 10 min. The thyroid tissues were stripped off the weasand, immersed in PBS, and cut into pieces (1–2 mm^3^) using sterile scissors and tweezers. The pieces were transferred into 50 mL centrifugation tubes, digested with collagenase II [2 mg/mL in DMEM/F12 medium (Sigma)] for 30–50 min at 30°C in a shaking water bath. The supernatants were filtered through a 200 μm mesh stainless steel sieve to disintegrate cell clots and remove matrix fragments. To collect all cells and estimate cell number, the suspension was centrifuged at 1,500 rpm for 10 min. The cells were suspended in complete medium (DMEM/F12 medium) supplemented with 10% (v/v) fetal calf serum (Thermo Scientific HyClone, Beijing, China), 10 μg/mL insulin, 5 μg/mL transferrin, 4 mM L-glutamine, 100 U/mL penicillin, and 100 μg/mL streptomycin (Sigma) (Föllmann et al., 2000). The cells were removed and placed into 25-cm^2^ flasks with a density of 2 × 10^6^ cells/flask, and the culture medium was refreshed every 2–3 days.

### Immortalization of Bovine Thyroid (BTY) Cells

Primary BTY cells were grown for 5–7 days and then passaged. The cells of passage 2 with 90% confluence were transfected with human TERT expression plasmid pCI-neo-hTERT (kindly provided by Dr. Tian Hong of the National Foot and Mouth Diseases Reference Laboratory, Lanzhou Veterinary Research Institute, Chinese Academy of Agricultural Sciences) using Lipofectamine 2000. After transfection for 24 h, the cells were passaged in the proportion of 1:10 and selected using G418 (400 μg/mL, Sigma) in complete medium. The culture medium was refreshed with complete medium supplemented with G418 every 3 days, and the cell debris and dead cells were removed. G418-resistant colonies were isolated by limited dilution 15 days later and cultured continually in complete medium supplemented with 200 μg/mL G418. One of the colonies was expanded and cultured by the series passage.

### Cell Proliferation Assay

The hTERT-BTY cells at passages 5, 10, 100, and 220 were seeded to 24-well plates at a density of 1 × 10^4^ cells/well. The cells were trypsinized every 24 h starting from 1 to 9 days after seeding and quantified using a hemocytometer.

### Gene Expression Analysis

Total RNA was extracted from primary BTY cells and passage-30 hTERT-BTY cells using TRIzol Reagent (Invitrogen). The complementary DNA synthesis reaction used anchored oligo (dT) 18 primers and the M-MLV reverse transcription kit (TaKaRa Bio, Japan). Primer 5.0 was used to design primers of the TSHR and NIS on the basis of the bovine gene sequence in the GenBank (TSHR Gene ID: 281553; NIS Gene ID: 505310). The primer sequences are given in **Table 2** and LA Taq (TaKaRa) was used in the PCR. The PCR protocol was as follows: initial denaturation at 94°C for 3 min then 35 cycles at 94°C for 30 s, 59.8°C for 30 s, and 72°C for 80 s; finally, an extension step at 72°C for 10 min. PCR products were analyzed by electrophoresis through 2% (w/v) agarose gel.

**Table 2:** Primers used for PCR amplification.

Gene	Sequences (5′→ 3′)
NIS-bovine-F-PCR	CCTCCAGGGCCGTGCTCATCAAC
NIS-bovine-R-PCR	GCCCCTCCCCTCCCCCATAACA
NIS-bovine-F-qPCR	CCTCTGCCATGACCACCATT
NIS-bovine-R-qPCR	CTGCTTTGTGGGGCACTTTC
TSHR-bovine-F-PCR	ATGGAGGGGCAGGGGATACG
TSHR-bovine-R-PCR	ATGCGCTGCTTCTAAGAGGAGTGC
TSHR-bovine-F-qPCR	GAGCTGTCTGTGTACACGCT
TSHR-bovine-R-qPCR	CTGAGCCTGGCGTTTACAGA
TSHR-Cricetulus-F-qPCR	CCATCCCCAGTCTGGCATTT
TSHR-Cricetulus-R-qPCR	CAGTGACACTGGTGGAGGAC
NIS-Cricetulus-F-qPCR	GTCCTCCAGGGCTCTTTCAC
NIS-Cricetulus-R-qPCR	AGATACAGGGGGTGGGTCTC
GAPDH-Cricetulus-F-qPCR	TTCACCACCATGGAGAAGGC
GAPDH-Cricetulus-R-qPCR	TGAAGTCGCAGGAGACAACC
NFKB2-bovine-F-qPCR	TTTTTCCCCTCCTCCTTGGC
NFKB2-bovine-R-qPCR	GCATCCACACTAGGCGTCTC
TRADD-bovine-F-qPCR	TTGGAGAATGCGCTCAGGAA
TRADD-bovine-R-qPCR	GGAGAAGGTGCCAGAGACTG
IRF3-bovine-F-qPCR	ACCCAGGAAGACACTCTGGA
IRF3-bovine-R-qPCR	CTCTGCAAGAACTGAGGGGG
DDX58-bovine-F-qPCR	TGTGGATGGTAGAGAAAGGTGC
DDX58-bovine-R-qPCR	GGCTAAGATTCTGGCTTTCTGG
TRAF2-bovine-F-qPCR	TGGAGAGCGAGCAGCAG
TRAF2-bovine-R-qPCR	TCGCCTGGCCTGAGGAG
CSF1-bovine-F-qPCR	GTTTTGTCTTCCGCCTGCTG
CSF1-bovine-R-qPCR	TCTGTGGCTCTTGATGGCTC
TNFRSF1B-bovine-F-qPCR	CACCACAAGATCCCAGCACA
TNFRSF1B-bovine-R-qPCR	GGGAACTTGGAGGACTTGGG
STAT1-bovine-F-qPCR	TCATGCCCAGAGAATCAGCC
STAT1-bovine-R-qPCR	CTGACTTTGCTGTCAAGCTCC
STAT2-bovine-F-qPCR	ACAGACAGGTGGATGAACTGC
STAT2-bovine-R-qPCR	CTGATGTGAACCCCAGACCC
DAXX-bovine-F-qPCR	GCGATGATGCAAGACAGGAA
DAXX-bovine-R-qPCR	GGCCCTCACCAGAATCCAAC
DUSP6-bovine-F-qPCR	CGACTCCTCCTCGGACATTG
DUSP6-bovine-R-qPCR	CCACAGGGAAAGAAGGCTGA
GAPDH-bovine-F-qPCR	CATGTTCCAGTATGATTCCACCC
GAPDH-bovine-R-qPCR	GAGCTTCCCGTTCTCTGCC


### Karyotype Analysis

Primary BTY cells and hTERT-BTY cells of passage 30 were treated with 0.1 μg/mL colchicine at 37°C for 6 h to obtain metaphase cells, which were harvested, and the numbers of chromosomes were determined. Individual pairs of chromosomes were identified using the G-banding technique according to size and space.

### Growth Characteristics of FMDV in Primary BTY and hTERT-BTY Cells

Foot-and-mouth disease virus strains of Asial/HN/06 (GenBank: KR073010.1), O/BY/CHA/2010 (GenBank: JN998085.1), A/GD/2013 (GenBank: KF450794.1), and A/WH/09 (GenBank: JF792355.1) were obtained from the National Foot and Mouth Diseases Reference Laboratory, China. Primary BTY and hTERT-BTY cells of passage 30 were grown for 2 days in six-well cell culture plates. When confluence reached 90%, they were infected with the virus diluted with MEM medium supplemented with 1% fetal bovine serum to provide an MOI of 0.001 plaque-forming units (PFUs)/cell. The cultures were kept at 37°C for 24 h and the typical CPE was observed by microscopy.

The distribution of FMDV in hTERT-BTY cells was detected with IFA. hTERT-BTY cells of passage 30 were infected or uninfected with FMDV strains of Asial/HN/06, O/BY/CHA/2010, A/GD/2013, and A/WH/09 (MOI = 0.001). The cells were fixed with 4% paraformaldehyde, permeablized with 0.1% Triton X-100 and blocked as described earlier. Then, the cells were incubated with guinea pig anti-FMDV serum (prepared by our laboratory, 1:100 dilution) for 12 h at 4°C. The cells were then incubated with goat anti-guinea pig IgG-FITC antibody (1:400 dilution) at 37°C for 1 h. The cell nuclei were stained with DAPI, and the cells were observed with a confocal microscope under a 100× magnification.

The qRT-PCR assay was used for detecting viral genome RNAs in infected primary BTY and hTERT-BTY cells of passage 50 at 0, 6, 12, 24, 36, 48, and 60 h post-infection. The cells were seeded in 24-well plates (5 × 10^5^ cells/well) for 24 h. Then the cell monolayers were infected with four FMDV strains at MOI 0.001. Viral genome RNAs were extracted using a Magna Pure LC Total Nucleic Acid Isolation Kit (Roche) with an automated robotic workstation (Roche) and detected using qRT-PCR every 6 h. qRT-PCR used the Platinum Quantitative RT-PCR ThermoScrip One-Step Mastermix Reagent Kit (Life Technologies, Camarillo, CA, United States). The PCR primer and probe were based on conserved sequences in the internal ribosomal entry site within the 5′-untranslated region of the FMDV genome (Reid et al., 2002). All cell samples were tested/evaluated in triplicate.

### Virus Titration

The titers of virus in hTERT-BTY cells, BTY cells at passage 3, and BHK-21 cells were determined using the TCID50 assay as previously described (Reed and Muench, 1938; Lian et al., 2016). Briefly, 10-fold serial dilutions from 10^-1^ to 10^-9^ of virus were inoculated into monolayer hTERT-BTY or BHK-21 cells in octuplicate in 96-well plates. After 72 h, the amount of pathogenic agent that caused a pathological change in 50% of cell cultures inoculated was determined using the Reed–Muench method.

### Transcriptome RNA-Seq

The differential expression of mRNA in infected and uninfected hTERT-BTY cells was detected to identify the pathways of innate immunity using RNA-seq. The hTERT-BTY cells were cultured for 24 h. The O/BY/CHA/2010 was inoculated with 0.001 PFU/cell. The samples were washed three times with PBS, and total RNA was extracted using TRIzol Reagent (Invitrogen, Carlsbad, CA, United States). The total RNA quantity and purity were analyzed using Bioanalyzer 2100 and RNA 6000 Nano LabChip Kit (Agilent, Santa Clara, CA, United States) with RIN number > 7.0. Approximately 10 μg of total RNA representing a specific adipose type was used to isolate Poly (A) mRNA with poly-T oligo-attached magnetic beads (Invitrogen). Then, the mRNA was fragmented into small pieces using divalent cations under elevated temperature, and the cleaved RNA fragments were reverse-transcribed to create the final cDNA library in accordance with the protocol for the mRNA Seq sample preparation kit (Illumina, San Diego, CA, United States). The average insert size for the paired-end libraries was 300 bp (±50 bp). Then, the paired-end sequencing was performed on an Illumina Hiseq 4000 (LC Sciences, United States) following the manufacturer’s recommended protocol. The Illumina paired-end RNA-seq approach was used to sequence the transcriptome, generating a million paired-end reads. This yielded gigabases (Gb) of the sequence. Prior to assembly, the low-quality reads were removed. A total of Gbp of cleaned, paired-end reads were produced. The raw sequence data was submitted to the NCBI Short Read Archive with an accession number. The reads of sample A and sample B were aligned to the UCSC^1^ *Bos taurus* sapiens reference genome using HISAT package. The mapped reads of each sample were assembled using StringTie. Then, all transcriptomes from the samples were merged to reconstruct a comprehensive transcriptome using Perl scripts. After the final transcriptome was generated, StringTie and Ballgown were used to estimate the expression levels of all transcripts. StringTie was used to determine the mRNA expression level by calculating FPKM. The differentially expressed mRNAs and genes were selected with log_2_ (fold change) > 1 or log_2_ (fold change) < –1 and with statistical significance (*P*-value < 0.05) using R package–Ballgown.

### q-PCR Assays

Relative quantitative real-time RT-PCR was used to analyze the expression of mRNA of various genes. The total RNA was extracted with TRIzol Reagent (Invitrogen). The RNA was reverse-transcribed into cDNA using random hexamer primers (TaKaRa) and the M-MLV reverse transcriptase (Promega). The SYBR Premix ExTaq kits (TaKaRa) were used in the quantitative PCR experiment, and the expression of glyceraldehyde-3-phosphate dehydrogenase (GAPDH) gene was determined as a reference gene. The primers used in the experiment are listed in **Table 2**. All the experiments were repeated at least three times, and relative mRNA expression levels were calculated using the threshold cycle (2^-ΔΔCt^) method.

### Statistical Analysis

Each experiment was performed in triplicate. Student’s *t*-test was performed using the SPSS 7.0 software. The level of significance is shown in figures.

## Ethics statement

Animal experiments were performed at Biosafety Level 3 laboratory of LVRI, Chinese Academy of Agricultural Sciences (Permission number: SYXK-GAN-2004-0005). All animal experiments were approved by the Gansu Animal Experiments Inspectorate and the Gansu Ethical Review Committee (License no. SYXK [GAN] 2010–003).

## Author Contributions

RM designed the study, conducted the experimental work, analyzed the data and wrote the manuscript. DS conducted the transcriptome analysis and modified the manuscript. FY provided constructed the recombinant viruses. ZZ, XL, and HZ provided ideals. HT provided assistance in the experimental work.

## Conflict of Interest Statement

The authors declare that the research was conducted in the absence of any commercial or financial relationships that could be construed as a potential conflict of interest.

## Footnotes

1 http://genome.ucsc.edu/
